# Meteorological and climatic variables predict the phenology of *Ixodes ricinus* nymph activity in France, accounting for habitat heterogeneity

**DOI:** 10.1038/s41598-022-11479-z

**Published:** 2022-05-12

**Authors:** Phrutsamon Wongnak, Séverine Bord, Maude Jacquot, Albert Agoulon, Frédéric Beugnet, Laure Bournez, Nicolas Cèbe, Adélie Chevalier, Jean-François Cosson, Naïma Dambrine, Thierry Hoch, Frédéric Huard, Nathalie Korboulewsky, Isabelle Lebert, Aurélien Madouasse, Anders Mårell, Sara Moutailler, Olivier Plantard, Thomas Pollet, Valérie Poux, Magalie René-Martellet, Muriel Vayssier-Taussat, Hélène Verheyden, Gwenaël Vourc’h, Karine Chalvet-Monfray

**Affiliations:** 1 Université de Lyon, INRAE, VetAgro Sup, UMR EPIA, 69280 Marcy l’Etoile, France; 2 Université Clermont Auvergne, INRAE, VetAgro Sup, UMR EPIA, 63122 Saint-Genès-Champanelle, France; 3 Université Paris-Saclay, AgroParisTech, INRAE, UMR MIA-Paris, 75005 Paris, France; 4 INRAE, Oniris, UMR BIOEPAR, 44300 Nantes, France; 5 Global Technical Services, Boehringer-Ingelheim Animal Health, 69007 Lyon, France; 6 Nancy Laboratory for Rabies and Wildlife, The French Agency for Food, Environmental and Occupational Health and Safety (ANSES), 54220 Malzéville, France; 7 Université de Toulouse, INRAE, UR CEFS, 31326 Castanet-Tolosan, France; 8 LTSER ZA PYRénées GARonne, 31326 Auzeville-Tolosane, France; 9 INRAE, UR EFNO, 45290 Nogent-sur-Vernisson, France; 10 ANSES, ENVA, INRAE, UMR 956 BIPAR, 94701 Maisons-Alfort, France; 11 INRAE, US 1116 AgroClim, 84914 Avignon, France; 12 INRAE, CIRAD, UMR ASTRE, 34398 Montpellier, France; 13 Present Address: Ifremer, RBE-SGMM-LGPMM, 17390 La Tremblade, France

**Keywords:** Ecological epidemiology, Ecological modelling, Entomology

## Abstract

*Ixodes ricinus* ticks (Acari: Ixodidae) are the most important vector for Lyme borreliosis in Europe. As climate change might affect their distributions and activities, this study aimed to determine the effects of environmental factors, i.e., meteorological, bioclimatic, and habitat characteristics on host-seeking (questing) activity of *I. ricinus* nymphs, an important stage in disease transmissions, across diverse climatic types in France over 8 years. Questing activity was observed using a repeated removal sampling with a cloth-dragging technique in 11 sampling sites from 7 tick observatories from 2014 to 2021 at approximately 1-month intervals, involving 631 sampling campaigns. Three phenological patterns were observed, potentially following a climatic gradient. The mixed-effects negative binomial regression revealed that observed nymph counts were driven by different interval-average meteorological variables, including 1-month moving average temperature, previous 3-to-6-month moving average temperature, and 6-month moving average minimum relative humidity. The interaction effects indicated that the phenology in colder climates peaked differently from that of warmer climates. Also, land cover characteristics that support the highest baseline abundance were moderate forest fragmentation with transition borders with agricultural areas. Finally, our model could potentially be used to predict seasonal human-tick exposure risks in France that could contribute to mitigating Lyme borreliosis risk.

## Introduction

Ongoing anthropogenic climate change impacts redistributions of vector populations and vector-borne diseases have become increasingly concerned as an alarming public health threat^1^. The effects of climate change on the population of *Ixodes ricinus* ticks (Acari: Ixodidae), the most important arthropod vector species in Europe, have become the topic of interest in this region^2^. As ixodid ticks are ectothermic vertebrates, their life cycles and key ecological processes, such as mortality, development rate, and host-seeking (questing) behaviour, are sensitive to weather conditions, particularly temperature and relative humidity^3,4^. Therefore, variations in these meteorological factors contribute to the regulation of the dynamics of tick abundance, phenology of questing activity, and eventually, human-tick exposure risks throughout the year. However, the direct impacts of climate change on tick population dynamics related to abiotic factors, such as temperature, which impacts the development rate^5^, and relative humidity, which impacts the mortality rate^6–8^, are not always straightforward. Rising temperatures could promote the probability of questing activity and interstadial developmental rate; however, ticks may also face a higher mortality risk at the same time^4^. Meanwhile, relative humidity influences the water regulation of ticks, affecting their questing behaviour and survival^6–8^. Furthermore, climate change could also pose indirect effects on tick populations through biotic factors, such as habitats^4^ and host populations (both density and behaviours)^2,9^. As a result, climate change has been expected to alter the distribution of tick populations, the activity of questing ticks, and human-tick exposure both geographically (expansions towards higher latitudes and altitudes)^10,11^ and seasonally (altered phenology)^12^.

*Ixodes ricinus* ticks have wide geographical and climatic ranges spanning all over Europe and some regions in northern Africa and western Asia^13^. Long-term observation of the questing activity of *I. ricinus* nymphs, for both phenology and abundance, has been an important indicator for the exposure risks to ticks and tick-borne diseases in these regions, particularly Lyme borreliosis^14–17^. *I. ricinus* ticks have been observed to exhibit different phenology (a seasonal variation) of questing activity across their wide range of geo-climatic distributions^18^, which determines the time of year when questing ticks are most or least likely to be encountered. The reported phenological patterns appeared to follow a climatic gradient across northern Africa to eastern Europe, with a unimodal pattern with a summer peak and a winter pause in freezing climates (Karelia, Russia), a bimodal pattern with spring and autumn peaks in intermediate climates (European Russia's temperate zone), and a unimodal pattern with a late winter peak in warm climates (Algeria)^18^. Meanwhile, the abundance (population size) of ticks in each area reflects the suitability/carrying capacity of the habitats for the populations, both land cover and climatic conditions^19^. Furthermore, when the suitability of the habitat is altered, long-term observations can reveal directional changes in questing activity over several years (inter-annual variation)^16^. However, such long-term observations that provide baseline data for evaluating how environmental changes will impact tick populations are currently lacking^20^. Therefore, to evaluates the effects of climate change on the distribution of tick-borne diseases, it is critical to understand the impacts of environmental factors, both abiotic and biotic, on all levels of the variations of tick populations and their questing activities over a long period of time across various geographical/climatic ranges.

However, most of the previous studies on the environmental effects on *I. ricinus* nymph activities in Europe have not captured both the temporal (seasonal and inter-annual) and spatial variations. Some studies focused on short-term observations at multiple sites, allowing for the comparison of abundance estimates but not seasonal and inter-annual variations^21–25^. While the other studies were long-term observations at a few sites, capturing seasonal and inter-annual variations and thus unable to compare with multiple sites^14–17^. Furthermore, the most widely used method for monitoring nymph activity, cloth-dragging sampling, has a low capture rate^26^. Therefore, this method produces a high level of uncertainty in abundance estimates across different environmental conditions and field investigators^27^, which makes comparing results from different observatories difficult.

France is a hotspot for *I. ricinus* and Lyme borreliosis^28^ and has a wide range of geographical and climatic characteristics^29^. An in-depth understanding of the characteristics of questing activity of *I. ricinus* across various geo-climatic areas is still needed to help control Lyme borreliosis in this area. The present study sought to investigate the activity of *I. ricinus* nymphs, both phenology and abundance, across a wide range of climatic region types in France over a long-term observation period. The sampling protocol suggested by a previous study was employed to reduce the uncertainty from the cloth-dragging method^27^. Also, this study aimed to assess the impacts of environmental factors, such as meteorological, bioclimatic, and habitat characteristics on the variations of *I. ricinus* nymph questing activity. Long-term investigations on nymph questing activity will help us better understand the effects of climate change on the dynamics of *I. ricinus* populations and eventually Lyme Borreliosis risk.

## Material and methods

### Sampling sites

**Figure 1:**
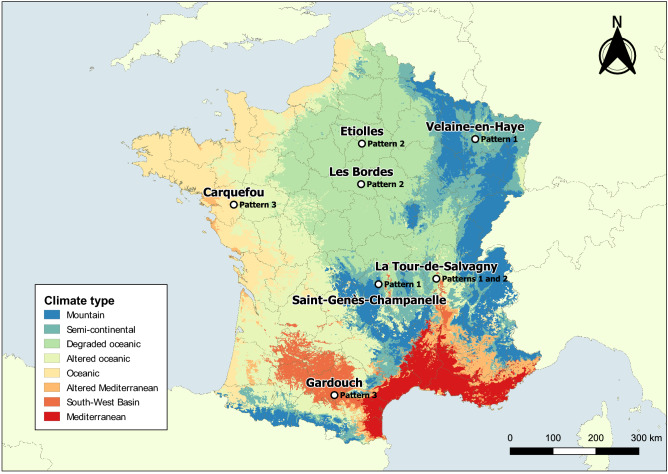
The distribution of tick observatories according to the climatic region types of continental France: (1) Etiolles (degraded oceanic); (2) Velaine-en-Haye (semi-continental); (3) Les Bordes (degraded oceanic); (4) Carquefou (oceanic); (5) La Tour de Salvagny (mixed); (6) Saint-Genès-Champanelle (mountain); (7) Gardouch (south-west basin). Phenological patterns observed at each observatory were also indicated. The map was created using QGIS version 3.8, Zanzibar (https://www.qgis.org). The climatic region types were previously classified by Joly et al.^29^.

Longitudinal observation campaigns for *I. ricinus* nymph activity were carried out at 11 sampling sites in forest areas from seven different tick observatories across France. Tick observatories are located at the following French municipal areas, where the coordinates of the centre of each municipal area and the climatic types^29^ are also provided as: (1) La Tour de Salvagny (45° 48′ 50.6″ N 4° 42′ 53.2″ E; Mixed climates); (2) Saint-Genès-Champanelle (45° 43′ 23.8″ N 3°01′ 08.0″ E; Mountain climate); (3) Etiolles (48° 37′ 59.9″ N 2° 28′ 00.1″ E; Degraded oceanic climate); (4) Carquefou (47° 17′ 58.5″ N 1° 29′ 26.0″ W; Oceanic climate); (5) Gardouch (43°23′ 25.7″ N 1° 41′ 02.1″ E; South-West Basin climate); (6) Velaine-en-Haye (48° 42′ 13.4″ N 6° 01′ 16.1″ E; Semi-continental climate); (7) Les Bordes (47° 48′ 47.3″ N 2° 24′ 01.3″ E; Degraded-oceanic climate) (Fig. 1). The observation campaigns were carried out from April/June 2014 to May/June 2021 in most observatories, except for Les Bordes, which began in April 2018.

Each tick observatory corresponds to one sampling site except La Tour de Salvagny, Gardouch, and Les Bordes (Table S1). In La Tour de Salvagny, we had to withdraw the observations at the original site (La Tour de Salvagny A) in September 2016 because the site became no longer accessible. In April 2017, we continued our observations at a nearby site, approximately 2 km apart (La Tour de Salvagny B). In Gardouch, the activity of questing nymphs was observed both inside and outside the enclosed area of an experimental station on roe deer (*Capreolus capreolus*), referred to as Gardouch Inside and Gardouch Outside, respectively. The estimated population density of roe deer in Gardouch Inside (50 individuals per 100 ha) was higher than Gardouch Outside (less than 20 individuals per 100 ha) (H. Verheiden, personal communication, 15th October 2021). Furthermore, three sampling sites in Les Bordes, approximately 1.2 km apart, were referred to as Les Bordes A, B, and C, respectively. Additional sampling sites of these observatories were considered and reported as distinct sampling sites in further analyses, resulting in a total to 11 sampling sites from 7 observatories. Furthermore, due to their geographical proximity, meteorological/climatic factors of different sampling sites from the same observatories were considered identical in subsequent statistical analyses, whereas land cover and topography factors could be varied.

Field observation campaigns were planned and carried out by local investigators who had been trained on the sampling protocol. The locations of forests, sampling sites, and passages were chosen where their biotopes are known to be suitable for *I. ricinus* tick populations around each observatory at the time the field observation campaigns started^30^. The observations were never carried out during the daytime when the weather was highly unfavourable to questing ticks, e.g., heavy rain, snow, or snow cover.

### Sampling protocol for questing *Ixodes ricinus* nymphs

Activity of questing *I. ricinus* nymphs was observed by a cloth-dragging sampling technique^31^. Within a 1-km radius, a 1 m × 1 m white cloth was dragged over 10 observation units of 10 m short-grass vegetative forest floors, called transects. For each transect, a repeated removal sampling design was used^27^. The cloth-dragging sampling process was successively repeated three times per sampling. All nymphs found on white cloth in each campaign were removed and collected in a vial for subsequent morphological identification^32^ by the same acarologists at the corresponding laboratories. As a result, the questing nymph activity of each sampling site was monitored as a total number of confirmed *I. ricinus* nymphs collected from three repeated sampling on 10 transects, equivalent to a surface area of 100 m^2^. This measure was considered as an indicator for tick abundance on the day of sampling. The same transects were repeatedly sampled throughout the study period at approximately 1-month intervals.

### Environmental data

We tested 28 environmental variables to explain the observed *I. ricinus* nymph activity (Table 1). These variables could be categorized as: (1) Daytime duration and meteorological variables (time-dependent, 9 variables); (2) Land cover, topography, and bioclimatic variables (time-independent, 19 variables).

**Table 1:** Environmental variables (meteorological, land cover, topography, and bioclimatic variables) used to explain *I. ricinus* nymph counts per 100 m^2^ in regression analysis.

Type	Group	Variable	Description
Meteorological	Temperature	${T}_{M}^{0:1}$	One-month moving average temperature; average temperature from 30 preceding days to the sampling day (°C)
		${T}_{M}^{3:6}$	Previous 3-to-6-month moving average temperature; average mean temperature of preceding 3–6 months (°C)
		${T}_{M}^{0:6}$	Six-month moving average mean temperature; between 6 preceding months to the sampling day (°C)
		${T}_{M}^{0:12}$	Twelve-month moving average mean temperature; between 12 preceding months to the sampling day (°C)
	Relative humidity	${U}_{N}^{0:1}$	One-month moving average minimum relative humidity; average value from 30 preceding days to the sampling day (%)
		${U}_{N}^{3:6}$	Previous 3-to-6-month moving minimum relative humidity of preceding 3–6 months (%)
		${U}_{N}^{0:6}$	Six-month moving average minimum relative humidity; between 6 preceding months to the sampling day (%)
		${U}_{N}^{0:12}$	12-month moving average minimum relative humidity; between 12 preceding months to the sampling day (%)
	Daytime	$Daytime$	Daytime duration (h)
Land cover, topography, and bioclimate^a^	Topography	${Mean}_{elv}$	Mean elevation (m)
		${sd}_{elv}$	Standard deviation of elevation (m)
		${p}_{flat}$	Proportion of flat area; slope ≤ 2.5%
		${p}_{north}$	Proportion of non-flat area facing north; aspect < 45° or ≥ 315°
		${p}_{east}$	Proportion of non-flat area facing east; 45° ≤ aspect < 135°
		${p}_{west}$	Proportion of non-flat area facing west; 135° ≤ aspect < 225°
		${p}_{south}$	Proportion of non-flat area facing south; 225° ≤ aspect < 315°
		$Catchment$	Catchment area
	Bioclimate	${BIO1}_{temp}$	Annual mean temperature (°C)
		${BIO2}_{diur}$	Mean diurnal range (°C)
		${BIO5}_{maxTemp}$	Maximum temperature of the hottest month (°C)
		${BIO12}_{prec}$	Annual precipitation (mm)
	Land cover	${H}_{CLC1}$	Shannon’s diversity index for level-1 CLC types
		${H}_{CLC2}$	Shannon’s diversity index for level-2 CLC types
		${H}_{Forest}$	Shannon’s diversity index for forest types
		${p}_{Forest}$	Percentage of forest-covering area (%)
		${n}_{Forest}$	Number of forest patches
		${ED}_{Forest}$	Forest edge density (m/km^2^)
	Soil	${pH}_{soil}$	Soil pH

^a^Land cover, topography, and bioclimatic variables were transformed by the principal component analysis as coordinates of PCA dimensions before being used in the regression analysis.

#### Daytime duration and meteorological variables

Daytime duration ($daytime$) from January 2013 to June 2021 at each sampling site was obtained from the corresponding latitude using *geosphere* package^33^. Hourly meteorological data (2-m temperature and relative humidity) were recorded locally at each forest. Subsequently, daily mean, minimum, and maximum values of temperature (${T}_{M}$, ${T}_{N}$, and ${T}_{X}$; in °C) and relative humidity (${U}_{M}$, ${U}_{N}$, and ${U}_{X}$; in %) were derived from these hourly records. The meteorological seasons of the temperate area in northern hemisphere are defined as: (1) Spring, 1st March to 31st May; (2) Summer, 1st June to 31st August; (3) Autumn, 1st September to 30th November; (4) Winter, 1st December to 28th or 29th February.

Missing values found on these local daily-level variables were imputed by the *random forest* algorithm in *mice* package^34^. External daily meteorological data, *i.e.*, daily average temperature and relative humidity, derived from neighbouring weather stations (Météo-France or INRAE), as well as month and year information, were used as auxiliary variables (Table S2). As a result, the imputation process creates a total of 500 iterated values for each variable. The median values of 500 imputations were used to replace the missing values.

The imputed daily meteorological data were subsequently used to calculate the averaged values in different lagged time intervals for further analysis, called interval-average variables^15^. The interval-average variables were generated to reduce the uncertainty that might arise during the imputation process and to capture the cumulative effects of the meteorological variables, which were mean temperature ${T}_{M}$ and minimum relative humidity ${U}_{N}$. The interval-average variables were defined as the average values of a meteorological variable $M\in$ {${T}_{M}$, ${U}_{N}$} during a period between ${t}_{1}$ to ${t}_{2}$ month(s) before the sampling, denoted as ${M}^{{t}_{1}:{t}_{2}}$, where 1 month consists of 28 days. As temperature conditions affect several ecological processes of tick populations, particularly developmental and questing rates^3^, the mean temperature ${T}_{M}$ was selected for further analysis to reflect the overall temperature effects. While the minimum relative humidity ${U}_{N}$ was chosen for the following reasons: (1) the survival of *I. ricinus* is highly sensitive to desiccation conditions^6–8^. As a result, when compared to mean or maximum relative humidity, minimum relative humidity is a relatively strong indicator of the effects of desiccation stress; (2) the variation of minimum relative humidity among all sites was higher than that of the mean and maximum relative humidity. This high variation allowed us to better describe meteorological characteristics of each sampling site.

Here, we hypothesized that interval-average meteorological conditions influence the dynamics of observed nymph activity at different time lags in different manners. Short-term lags may have an impact on immediate responses, such as the probability of questing. At the same time, long-term lags may influence the dynamics of nymph abundance, which is associated with development and survival rates. Therefore, we explored the impact of each meteorological variable at following time lags on the observed nymphs activity in subsequent regression analysis: (1) 1-month moving average condition, ${M}^{0:1}$; (2) previous 3-to-6-month moving average condition, ${M}^{3:6}$; (3) 6-month moving average condition, ${M}^{0:6}$; (4) 12-month moving average condition, ${M}^{0:12}$. For instance, ${T}_{M}^{0:1}$ denotes 1-month moving average temperature, representing an average of temperature between 0 and 1 months (0–28 days) before the day of sampling.

In addition to the interval-average variables, monthly and seasonal average values of mean temperature and minimum relative humidity during the observation period were also calculated to describe the characteristics of meteorological conditions of each sampling site.

#### Land cover, topography, and bioclimatic variables

We obtained land cover, topography, and bioclimatic data from a 1-km radius buffer area around the center of each sampling site to capture habitat characteristics across all 10 transects. All the variables were handled and obtained by using QGIS version 3.8.0^35^. The digital elevation model (DEM) data derived from the Shuttle Radar Topography Mission (SRTM) database^36^ was used to describe the topographic features of sampling sites, which included the mean (${mean}_{elv}$) and standard deviation (${sd}_{elv}$) of the elevation (in m above sea level), the proportion of flat area (${p}_{flat}$; defined by the slope ≤ 2.5%^37^), the proportion of area facing north (${p}_{north}$), east (${p}_{east}$), west (${p}_{west}$), and south (${p}_{south}$), and the catchment area ($catchment$) as a proxy variable for moisture. Bioclimatic variables for each site (historical average conditions during 1970–2000) were derived from the WorldClim database^38^, including the annual mean temperature (${BIO1}_{Temp}$; in °C), the mean diurnal range (${BIO2}_{Diur}$; in °C), the maximum temperature of the warmest month (${BIO5}_{maxTemp}$; in °C), and the annual precipitation (${BIO12}_{Prec}$; in mm). The land cover features of each sampling site were described using the CORINE Land Cover (CLC) 2018^39^, while the characteristics of forests were explained by the BD forêt version 2 data^40^. The forest fragmentation was characterized by the percentage of forest-covering area (${p}_{Forest}$), the forest edge density (${ED}_{Forest}$; in m/km^2^), and the number of forest patches (${n}_{Forest}$). While the diversities of the land cover types (level-1 and level-2 CLC) and the forest types were calculated by using the Shannon’s diversity index^41^ ($H$) as $H=\sum_{i=1}^{S}{p}_{i}\mathrm{ln}{p}_{i}$, where $S$ is the total number of land cover/forest types and ${p}_{i}$ is the proportion of land cover/forest type $i$ within the 1-km radius buffer area. The Shannon’s diversity index for level-1 CLC, level-2 CLC, and forest types were denoted as ${H}_{CLC1}$, ${H}_{CLC2}$, and ${H}_{Forest}$, respectively. Finally, the soil pH data (${pH}_{soil}$) was retrieved from the European Soil Data Centre (ESDC) database^42^.

### Statistical analysis

All the statistical analyses were carried out using the programming language R version 3.6.0^43^. The variations of questing nymph population of each site were described by using (1) baseline annual nymph counts (spatial variation); (2) phenological patterns (seasonal variation). A baseline annual nymph count of site $i$ (${{N}_{base}}_{i}$) was defined as a summation of monthly median nymph counts ${\varvec{\tilde{N}}}_{i}=\{{\tilde{N }}_{i,t}\}$ across all 12 months $t\in \left\{\mathrm{1,2},\dots ,12\right\}$ and expressed as: ${{N}_{base}}_{i}=\sum_{t=1}^{12}{\tilde{N }}_{i,t}$. Subsequently, the monthly median nymph counts of each site ${\varvec{\tilde{N}}}_{i}$ were transformed into normalized monthly median nymph counts ${\varvec{\tilde{N}}}_{i}^{*}=\{{\tilde{N }}_{i,t}^{*}\}$ following Eq. (1) to have a range value of 0 to 1, which allows us to compare phenological patterns among all sites that have different annual baseline nymph counts.

1 $${\tilde{N }}_{i,t}^{*}=\frac{{\tilde{N }}_{i,t}}{\mathrm{max}({\stackrel{\sim }{{\varvec{N}}}}_{i})}$$

The term $\mathrm{max}({\stackrel{\sim }{{\varvec{N}}}}_{i})$ denoted the maximum monthly median nymph counts. The normalized median nymph count ${\tilde{N }}_{i,t}^{*}$ of 1 indicates the maximum nymph activity (peak), while the value ${\tilde{N }}_{i,t}^{*}$ of 0 designates the absence of nymph activity. Afterwards, the phenological patterns were descriptively classified using the following criteria: (1) the season which the peaks of activity arrive; (2) evidence of reduced activity during winter (November–January); (3) the number of activity waves in a year, whether the pattern is unimodal or bimodal. After assigning phenological patterns to each site, the overall trends of different patterns were derived from medians of the normalized monthly median nymph count ${\tilde{N }}_{i,t}^{*}$ from all sites that belonged to each pattern. Furthermore, the directional changes in the maximum nymph counts were tested using a Spearman’s rank correlation coefficient, a *p*-value < 0.05 was considered significant.

The effects of environmental variables (meteorological, land cover, topographical and bioclimatic variables) on the abundance of *I. ricinus* nymphs, were evaluated by a multivariate mixed-effects negative binomial regression, using the *glmmTMB* package^44^. Recalled that the negative binomial distribution is a generalized form of the Poisson distribution with an additional shape parameter $\theta$ that allows the variance to be independent to the mean $\mu$. The number of *I. ricinus* nymphs per 100 m^2^ was considered the response variable, and the sampling site served as the random effects for intercepts as displayed in Eq. (2):

2 $$\begin{aligned} & N_{i} \sim NegBinom(\mu_{i} ,\theta ) \\ & {\text{ln}}\;\mu_{i} = \alpha_{0} + \alpha_{i} + X_{i} \beta \\ \end{aligned}$$

where ${N}_{i}$ denoted nymph counts per 100 m^2^ of site $i$, ${\mu }_{i}$ and $\theta$ represented mean of site $i$ and the shape parameter of the negative binomial distribution, respectively. ${\alpha }_{0}$ was an intercept of the regression model, while ${\alpha }_{i}$ was the random intercept of site $i$. Finally, ${X}_{i}$ and $\beta$ denoted a covariate vector of site $i$ and a vector of regression coefficients for fixed effects, respectively.

The multicollinearity among all environmental variables was evaluated using Pearson’s correlation coefficient $r$. Highly correlated variables, with $\left|r\right|$ ≥ 0.6, were not included in the same model. The principal component analysis (PCA) was used to reduce the dimensions of multiple highly correlated land cover, topography, and bioclimatic variables, using *FactoMineR*^45^ and *factoextra*^46^ packages. To reduce the number of variables considered in the regression analysis, the coordinates of individual sampling sites on the PCA dimensions were subsequently explored as explanatory variables. Non-linearity and interaction effects of the fixed effects were also investigated.

Multivariable models were explored by both forward and backward selection of significant variables decreasing the Akaike information criterion (AIC). Variations of the data explained by fixed effects were monitored by a pseudo-R^2^ statistic using the *MuMIn* package^47^. Residuals of the models were evaluated using the *DHARMa* package^48,49^. The performance of the best-fitted model was assessed by a resampling method. Briefly, 50% of the original dataset was randomly sampled to fit with the best model for 500 iterations, referred to as resampling models. The distributions of regression coefficients across 500 resampling models were compared against those of the model with the original dataset, called the complete model. Additionally, the coverage probability (CP) of each explanatory variable, defined as the percentage of resampling models that produced regression coefficients that were significantly different from zero ($p$ < 0.05), were also calculated. The CP index is a measure of how robust each variable is to explain observed data. Finally, the effects of each environmental variable were predicted using the *effects* package^50^.

## Results

### Characteristics of sampling sites

**Figure 2:**
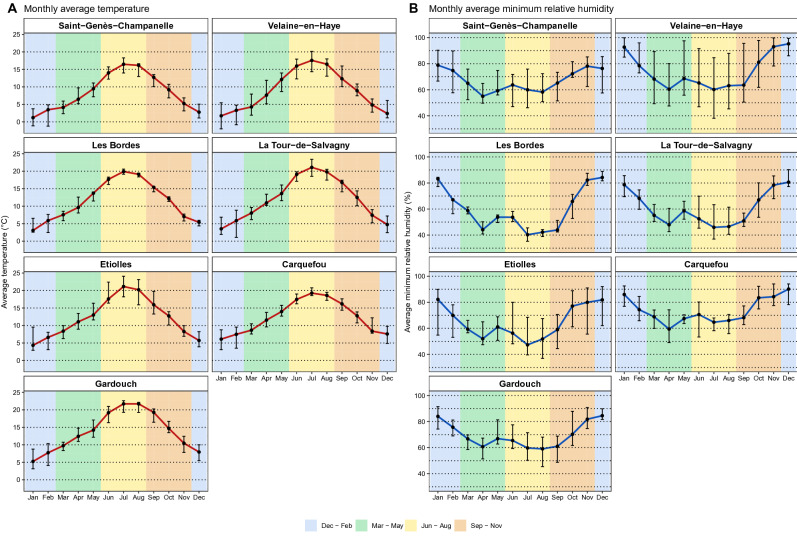
Monthly average meteorological conditions of 7 tick observatories: (**A**) average temperature ${T}_{M}$; (**B**) minimum relative humidity ${U}_{N}$. The average values were calculated using the imputed data from January 2013 to June 2021, with the exception of Les Bordes, which was calculated from January 2017 to June 2021. The points represented the median of the monthly average values over the observed period, while the error bars represent the maximum and minimum monthly average values. Background colours indicated meteorological seasons of the temperate areas of northern hemisphere: Spring, 1st March to 31st May (green); Summer, 1st June to 31st August (yellow); Autumn, 1st September to 30th November (brown); Winter, 1st December to 28th or 29th February (blue).

During 2014 to 2021, the activity of questing *I. ricinus* nymphs was observed from 11 sample sites at 7 tick observatories in France. The characteristics of each sampling site, i.e., meteorological, land cover, topography, and bioclimatic features, appeared to be highly correlated (Fig. S1). According to the meteorological conditions (Fig. 2, Figs. S2, S3A,B), the characteristics of tick observatories can be classified as: (1) cold average temperature with the average winter temperature < 2.5 °C (Saint-Genès-Champanelle and Velaine-en-Haye); (2) warm average temperature with average winter temperature ~ 7 °C and high summer humidity with average minimum relative humidity in summer > 60% (Gardouch and Carquefou); (3) intermediate average temperature with average winter temperature between 4 and 6 °C and low summer humidity with average minimum relative humidity in summer < 55% (La Tour de Salvagny, and Etiolles).

**Figure 3:**
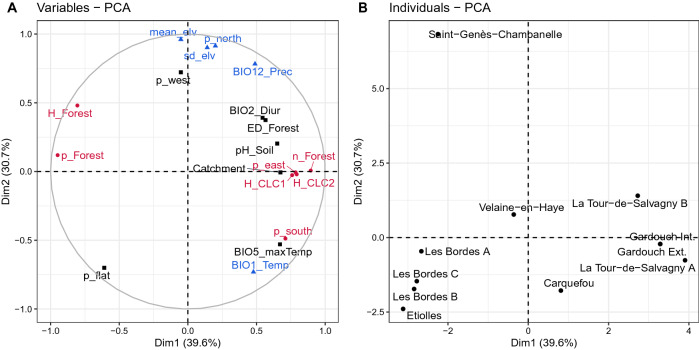
Principal component analysis (PCA) for land cover, topography, and bioclimate characteristics of all sampling sites: (**A**) PCA plot for land cover, topographical, and bioclimatic variables. Colours indicate whether the variable contributes more to Dimension 1 (red), Dimension 2 (blue), or other higher dimensions (black), using the cumulative contribution of ~ 60%; (**B**) PCA plot for individual sampling sites. Coordinates of the individual PCA plot were subsequently used in the regression analysis.

The land cover, topography, and bioclimatic characteristics of each sampling site were described in Tables S3–S5, respectively. The first two dimensions of the principal component analysis (PCA) explained 70.3% of the variation in the land cover, topography, and bioclimatic variables (Fig. 3A and Fig. S4). The first dimension differentiated each sampling site predominantly based on forest and land cover characteristics, in which the first 4 contributions were the number of forest patches ${n}_{Forest}$, percentage of forest-covering area ${p}_{Forest}$, Shannon’s diversity index for forest types ${H}_{Forest}$, and Shannon’s diversity index for level-2 CORINE Land Cover (CLC) types ${H}_{CLC2}$, respectively (Fig. 3A and Fig. S4). The Shannon’s diversity index for forest types ${H}_{Forest}$ was found to be negatively correlated with the Shannon’s diversity index for the CLC types, ${H}_{CLC1}$ and ${H}_{CLC2}$, (Fig. S1). The sampling sites in La Tour de Salvagny A, La Tour de Salvagny B, Gardouch Inside and External were relatively high fragmented forests, less percentage of forest-covering areas, more diverse in the CORINE Land Cover types, and less diverse in the forest types. While the sampling sites in Saint-Genès-Champanelle, Les Bordes and Etiolles were covered by a high percentage of forest areas with less forest fragmentation and high diversity in forest types.

The second PCA dimension of was driven by topographic and bioclimatic features, such as mean ${mean}_{elv}$ and standard deviation ${sd}_{elv}$ of elevation, proportion of non-flat area facing north ${p}_{north}$, annual precipitation ${BIO12}_{prec}$, and annual mean temperature ${BIO1}_{Temp}$ (Fig. 3A and Fig. S4). The sampling site in Saint-Genès-Champanelle was located at a high uneven elevation above the sea level (mountainous) with higher annual precipitation and lower annual temperature compared to all other sampling sites. While sampling sites of Les Bordes and Etiolles were located at flat areas with the lowest annual precipitation. The coordinates for each sampling site on the first and second dimensions on the PCA plot (Fig. 3B) were used to describe land cover, topography, and bioclimatic characteristics in subsequent regression analysis, denoted as $Dim1$ and $Dim2$, respectively. In addition, soil in the forest of Les Bordes appeared to be acidic, with an estimated pH of 4 (Table S3).

### Abundance indicators of questing *Ixodes ricinus* nymphs

**Figure 4:**
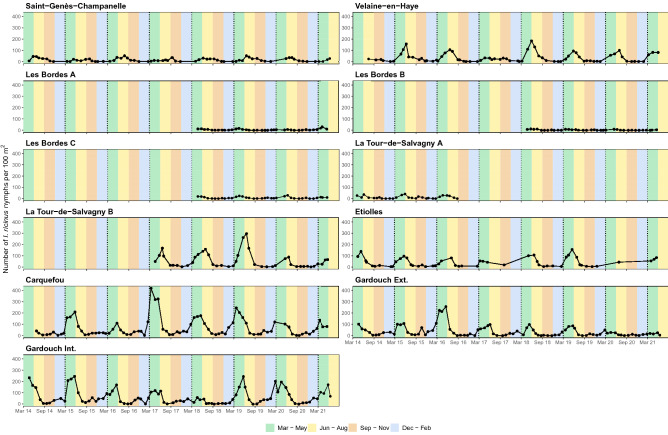
Number of *Ixodes ricinus* nymphs per 100 m^2^ obtained from 11 sampling sites. Background colours indicate meteorological seasons of temperate areas of the northern hemisphere: Spring, 1st March to 31st May (green); Summer, 1st June to 31st August (yellow); Autumn, 1st September to 30th November (brown); Winter, 1st December to 28th or 29th February (blue). Vertical dashed lines indicate 1-year interval.

**Figure 5:**
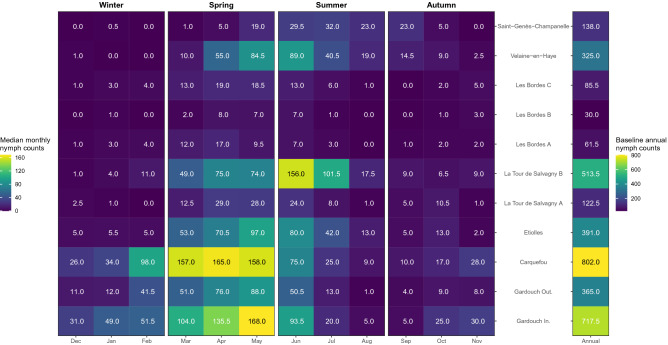
Monthly median nymph counts (left), and baseline annual nymph counts (right) obtained from 11 sampling sites. Meteorological seasons of temperate areas of the northern hemisphere: Spring, 1st March to 31st May; Summer, 1st June to 31st August; Autumn, 1st September to 30th November; Winter, 1st December to 28th or 29th February.

During the observation period, 631 campaigns were carried out, yielding a total of 22,912 *I. ricinus* nymphs (Table S1). Since the study design did not allow us to estimate the true abundance of questing nymphs, this study reported confirmed *I. ricinus* nymph counts, monthly median nymph counts, and baseline annual nymph count as indicators of the true abundance (Figs. 4, 5, and Fig. S3C). The baseline annual nymph count was highest in Carquefou (802 per 100 m^2^), Gardouch Inside (717.5 per 100 m^2^), and La Tour de Salvagny B (513.5 per 100 m^2^), while it was lowest in Les Bordes A to C (30–85.5 per 100 m^2^), La Tour de Salvagny A (122.5 per 100 m^2^), and Saint-Genès-Champanelle (138 per 100 m^2^) (Fig. 5).

### Phenological patterns of questing *Ixodes ricinus* nymphs

**Figure 6:**
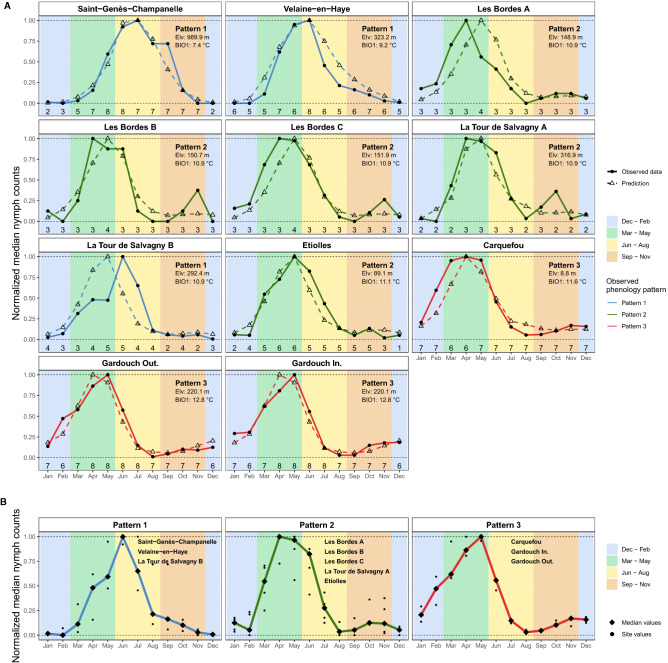
Phenological patterns of *I. ricinus* nymph activity observed at 11 sampling sites: (**A**) observed and predicted normalized monthly median nymph counts of each sampling site. The value of 1 indicates the maximum nymph activity (peak), while the value of 0 indicates the absence of nymph activity. Line types distinguish between the observation (solid) and prediction from the best-fitted model (dashed). Line colours indicate different phenological patterns of the observed data: Pattern 1, a unimodal pattern with a summer peak and a winter pause (blue); Pattern 2, a bimodal pattern with a greater activity peak in spring and a smaller peak in autumn (green); Pattern 3, a unimodal pattern with a spring peak without winter pause (red). Text annotations demonstrate mean elevation above the sea level ${mean}_{elv}$ (m) and average annual temperature ${BIO1}_{temp}$ (°C). Numbers at the bottom represent the number of observations of each month throughout the study period; (**B**) normalized monthly median nymph counts observed at each site (circle) and the overall trend of each phenological pattern (diamond and line). The overall trend of each pattern was determined using the medians of the values from all sites that showed that pattern, as indicated by text annotations. Background colours indicate meteorological seasons of the temperate areas of northern hemisphere: Spring, 1st March to 31st May (green); Summer, 1st June to 31st August (yellow); Autumn, 1st September to 30th November (brown); Winter, 1st December to 28th or 29th February (blue).

The normalized monthly median nymph counts revealed three phenological patterns of questing nymph activity observed among the 11 sampling sites (Fig. 6 and Fig. S3D): (1) Pattern 1: a unimodal pattern with a relatively long peak activity beginning in late spring, reaching its maximum in summer and fading out in autumn, with no or very low activity in winter (Saint-Genès-Champanelle, Velaine-en-Haye, and La Tour de Salvagny B); (2) Pattern 2: a bimodal pattern with an earlier nymph activity reaching its peak in spring (the first peak), fading out in summer, reappearing in autumn (the second peak), and fading out again in winter (Les Bordes A to C, La Tour de Salvagny A, and Etiolles); (3) Pattern 3: a unimodal pattern with moderate nymph activity throughout autumn and winter, reaching its peak in spring, and fading out quickly in summer (Carquefou, Gardouch Inside, and Gardouch Outside). Interestingly, questing nymphs have also been observed during winter in most sites. In addition, a decreasing trend in annual maximum nymph counts was observed more evidently in Gardouch Outside (Spearman’s rank correlation coefficient = − 0.8982; *p*-value < 0.05), while no apparent directional change was observed in other sites (Fig. 4 and Fig. S5).

### Environmental impacts on nymphs activity

Several mixed-effects negative binomial regression models were evaluated to explain impacts of environmental factors on observed *I. ricinus* nymph counts. The best-fitted model was selected by the AIC value, while other model characteristics were compared by dispersion parameter, variance of the random intercepts, and pseudo-R^2^ statistics (Table 2). Fixed effects in Model 5, predominantly meteorological variables, explained 65.1% of the total variance of nymph counts.

**Table 2:** The Akaike information criterion (AIC), dispersion parameter of the negative binomial regression, and the variance of the intercepts when sampling sites are considered a random effect.

Model	Description	AIC	Dispersion parameter	Variance of intercepts	Pseudo-R^2^
0	Null	5447.6	0.70449	1.0057	0.0000
1	$({T}_{M}^{0:1}{)}^{3}+({T}_{M}^{0:1}{)}^{2}+{T}_{M}^{0:1}$	5272.6	0.92771	1.0004	0.2495
2	$({T}_{M}^{0:1}{)}^{3}+({T}_{M}^{0:1}{)}^{2}+{T}_{M}^{0:1}*{BIO1}_{temp}$	5256.5	0.95624	0.9707	0.2729
3	$({T}_{M}^{0:1}{)}^{3}+({T}_{M}^{0:1}{)}^{2}+{T}_{M}^{0:1}*{BIO1}_{temp}+ ({T}_{M}^{3:6}{)}^{3}+({T}_{M}^{3:6}{)}^{2}+{T}_{M}^{3:6}$	4858.0	1.98588	0.9814	0.617
4	$({T}_{M}^{0:1}{)}^{3}+({T}_{M}^{0:1}{)}^{2}+{T}_{M}^{0:1}*{BIO1}_{temp}+ ({T}_{M}^{3:6}{)}^{3}+({T}_{M}^{3:6}{)}^{2}+{T}_{M}^{3:6}+({U}_{N}^{0:6}{)}^{3}+({U}_{N}^{0:6}{)}^{2}+{U}_{N}^{0:6}$	4823.9	2.13044	1.0004	0.6406
5	$({T}_{M}^{0:1}{)}^{3}+({T}_{M}^{0:1}{)}^{2}+{T}_{M}^{0:1}*{BIO1}_{temp}+ ({T}_{M}^{3:6}{)}^{3}+({T}_{M}^{3:6}{)}^{2}+{T}_{M}^{3:6}+({U}_{N}^{0:6}{)}^{3}+({U}_{N}^{0:6}{)}^{2}+{U}_{N}^{0:6}+(Dim1{)}^{3}+(Dim1{)}^{2}+Dim1$	4811.9	2.13050	0.4224	0.6507

Asterisks indicated the interaction terms between 2 variables.

Model diagnostics showed no indication of lack of fit (Kolmogorov–Smirnov test: *p*-value = 0.2067), under- or over-dispersion in the residuals (*p*-value = 0.08), or residual outliers (*p*-value = 1) (Fig. S6). The strength of the best-fitted model was assessed by resampling half of the data to fit with this model. The regression coefficients yielded from 500 resampling models were similar to those of the complete model with mostly high coverage probability (Fig. S7 and Table S6).

**Figure 7:**
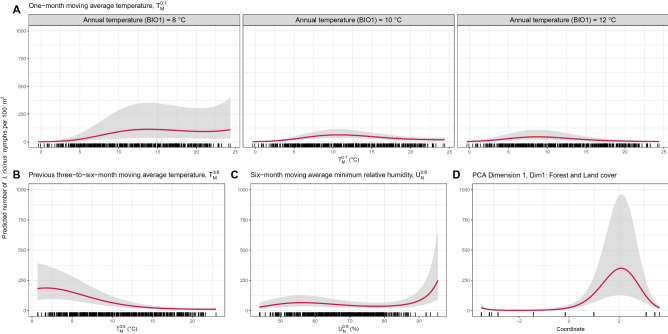
Predicted effects of environmental factors on the number of *I. ricinus* nymphs per 100 m^2^ given the average values of all other variables: (**A**) 1-month moving average temperature, an averaged mean temperature between previous 30 days and sampling day (${T}_{M}^{0:1}$) with an interaction effect with annual temperature (${BIO1}_{Temp}$); (**B**) previous 3-to-6-month moving average temperature, an averaged mean temperature between 3 and 6 months prior to the sampling day (${T}_{M}^{3:6}$), (**C**) 6-month moving average minimum relative humidity, an averaged minimum relative humidity between previous 6 months and the sampling day (${U}_{N}^{0:6}$); (**D**) coordinates on Dimension 1 of the principal component analysis ($Dim1$), representing forest and land cover characteristics. Gray shaded areas indicate a 95% confidence interval. Rugs display the distribution of observed values.

The best-fitted model (Model 5) suggested that phenology of *I. ricinus* nymphs non-linearly responded to meteorological factors, such as 1-month moving average temperature ${T}_{M}^{0:1}$, previous 3-to-6-month moving average temperature ${T}_{M}^{3:6}$, and 6-month moving average minimum relative humidity ${U}_{N}^{0:6}$. The interaction effect between the 1-month moving average temperature ${T}_{M}^{0:1}$ (weather) and the annual average temperature ${BIO}_{Temp}$ (climate) explained that the nymph counts observed at different climates arrive at peaks at different 1-month moving average temperatures, where nymph counts in warmer climates peaked at lower 1-month moving average temperatures (Fig. 7A). Also, the best-fitted model explained that nymph counts were highest at low previous 3-to-6-month moving average temperatures ${T}_{M}^{3:6}$ (Fig. 7B) and high 6-month moving average minimum relative humidity ${U}_{N}^{0:6}$. (Fig. 7C). In addition, it should be noted that because the daytime duration was highly correlated with several meteorological variables, such as 1-month moving average temperature ${T}_{M}^{0:1}$, previous 3-to-6-month moving average temperature ${T}_{M}^{3:6}$, and 1-month moving average minimum relative humidity ${U}_{N}^{0:1}$ (Fig. S1); therefore, it was excluded from the best-fitted model.

The Dimension-1 PCA coordinates $Dim1$, representing the forest and land cover characteristics of different sampling sites, markedly reduced the variance of random intercepts from 1.0004 in Model 4 to 0.4224 in Model 5 (Table 2). Thus, the forest and land cover characteristics in Model 5 significantly explained model intercepts which indicates their impacts on the baseline nymph count levels. The landscape that supports more baseline nymph counts was suggested to have a moderate percentage of forest-covering areas ${p}_{Forest}$, number of forest patches ${n}_{Forest}$, and Shannon’s diversity index for level-1 (${H}_{CLC1}$) and level-2 CORINE Land Cover types (${H}_{CLC2}$) (Fig. 7D). It should be noted that the Dimension-2 PCA coordinates $Dim2$, driven mainly by the mean and standard deviation of the elevation above sea level, was not significant and therefore not included in the final model.

Finally, the prediction from the best-fitted model replicated well the phenological patterns of observed *I. ricinus* nymphs (Fig. 6 and Fig. S8), particularly sampling sites with more observations, i.e., Saint-Genès-Champanelle, Velaine-en-Haye, Etiolles, Carquefou, Gardouch Inside, and Gardouch Outside. However, the autumn peaks of the phenological Pattern 2 (Fig. 6) and the interannual variations of the nymph counts (Fig. S8) remain imperfectly predicted by the model using meteorological variables.

## Discussion

The variations in tick activity across space and time can be classified into four levels: (1) baseline abundance variation; (2) sub-daily variation; (3) seasonal variation; (4) inter-annual variation. The baseline abundance reflects the suitability/carrying capacity of the habitats for the populations, both land cover and climatic conditions^19^. The sub-daily variation arises from the immediate responses of questing ticks to rapid microenvironment changes, such as local temperature, relative humidity^51^, solar altitude, or presence of dew drops, to maintain their water balance^52^. Such responses reflect the instantaneous probability of questing. While seasonal and inter-annual variation reflects the long-term cumulative results of the questing behaviour as well as reproduction, development, and mortality rates on the tick population^3^. However, previous studies on the effects of the environment on *I. ricinus* nymph activities in Europe did not capture all the aforementioned levels of variation. They largely focused on either a short-term period for multiple sites^21–24^ or a long-term period for a few areas^14–17^. The present study attempted to explore the baseline abundance, seasonal, and inter-annual variations of *I. ricinus* nymph activity by expanding the observation across a wide range of geographical/climatic areas over a long period, involving a total of 11 sampling sites from 7 tick observatories in France over 8 years. With the repeated removal sampling design, we controlled the uncertainty on the proxy measure of tick abundance (nymph counts per 100 m^2^) that arises from the variability of sampling rate across different sites and times^27,53^. Furthermore, all transects were covered with low vegetation, which have been shown to maximize sampling rates compared to other types of vegetation floors^27^, resulting in higher accuracy in estimating the proxy of tick abundance. To avoid the uncertainty caused by the variations of the environment within a sampling site, we repeated tick sampling from the same transects throughout the observation period. Furthermore, we strived to minimize the interference of tick populations and their environment during the long-term observation period by using a 1-month sampling interval. As all of the transects were open areas, ticks were free to relocate in and out of the transects, either directly or through hosts, and compensate for the monthly removals of tick populations. Nevertheless, the sub-daily variations in questing behaviour or immediate response to meteorological conditions among nymphs from different regions were not captured by this study design.

Furthermore, the activity of *I. ricinus* in other life stages (larvae and adults) was suggested to respond differently to the environment than the nymph stage, depending on their population cohorts^54^. However, the protocol and performance of sampling methods for estimating tick abundance were well described only for the nymph stage^27,31^, and to our knowledge, no studies have proposed a standard protocol and assessed the performance of the sampling method for larva and adult stages of *I. ricinus*. As a result, the sampling method used in our study did not allow us to observe the activity of larvae and adults. To investigate potential environmental effects on the activity of these stages, more research is needed on the performance of sampling protocols for larvae and adults that give us accurate abundance estimates and allow us to compare several environmental conditions.

Phenological patterns of *I. ricinus* nymph activity observed in our study tended to be driven by climatic characteristics. In general, nymph activity was markedly reduced during two types of unfavourable temperature conditions: (1) cold conditions with monthly average temperature < 3 °C; (2) hot conditions with monthly average temperature > 16.5 °C. Temperature conditions in mountainous climate (Saint-Genès-Champanelle and Velaine-en-Haye) were intensely unfavourable in winter, resulting in a near-complete halt. In contrast, conditions in summer were not as severe, resulting in a reduction in nymph activity arriving in mid-to-late summer. As a result, a unimodal pattern with a summer peak and complete winter pause was observed in this climate (Pattern 1). However, winter temperatures were relatively benign in the oceanic (Carquefou) and south-west basin (Gardouch) climates, allowing nymph activity to increase. Meanwhile, unfavourable conditions were severe during the summer, resulting in a sharp decrease in nymph activity in early summer, and therefore, a unimodal pattern without a winter pause (Pattern 3). In intermediate climates (Les Bordes and Etiolles), winter brought unfavourable conditions though not as severe as in mountainous climates, resulting in a reduction in activity but not complete cessation. While summer also brought unfavourable conditions, resulting in a significant decrease in activity from early to mid-summer, and thus a spring–autumn bimodal pattern (Pattern 2). However, despite the phenological patterns 1 and 3 being clearly distinct, we found that the existence of the autumn peaks in pattern 2 was ambiguous for the following reasons: (1) most sites with phenological pattern 2 had a low baseline abundance level. One additional nymph count due to variability of the sampling could lead to a significant false signal for the autumn activity; (2) compared to other patterns, tick activity at most sites with phenological pattern 2 was observed for a shorter period (a smaller sample size). A longer observation period is required to confirm the presence of bimodal patterns at these sites. Furthermore, we observed different phenological patterns at La Tour de Salvagny A and B, despite these two sites being approximately 2-km apart. The heterogeneity of the phenological patterns could have arisen from the following reasons: (1) THE phenological pattern 2 observed from a short-term period at La Tour de Salvagny A was ambiguous, as previously discussed; (2) different landscapes and host densities in these sites influenced differently tick population dynamics and apparent tick activities.

The overall phenological patterns observed in our study followed a climatic gradient previously reported: (1) a unimodal pattern with a summer peak and a winter pause (Pattern 1) in freezing climates (Karelia, Russia); (2) a bimodal pattern with spring–autumn peaks (Pattern 2) in intermediate climates (European Russia's temperate zone); (3) a unimodal pattern with a late winter peak (Pattern 3) in warmer climates (Algeria)^18^. Also, a similar phenology trend observed in one mountain region in Switzerland was reported to follow an elevation gradient where the spring–autumn bimodal phenology (Pattern 2) shifted to a late-spring unimodal phenology (Pattern 1) at a higher elevation^17^, presumably due to a temperature gradient. A similar climatic trend was observed in *Ixodes scapularis* nymphs in a study conducted across a large geographic area in the United States, with peak nymph activity occurring in the summer in colder regions, and late spring in warmer regions^55^. However, the climatic gradient of phenological patterns observed in our study might not apply to all *I. ricinus* populations. For example, an *I. ricinus* population in a freezing climatic area (Finland) has been reported to have a spring–autumn bimodal phenology (Pattern 2)^56^, rather than a summer unimodal phenology (Pattern 1) as suggested by our results. Further, *I. ricinus* populations from different climates were reported to begin their host-seeking behaviour at different temperature thresholds, either through local adaptation or phenotypic plasticity^57,58^, which may result in different phenological patterns.

However, according to the climatic gradient observed in our study, it is conceivable to hypothesize that a gradual shift in climate due to climate change towards warmer temperature and lower relative humidity will result in the following phenological changes to *I. ricinus* tick populations in France: (1) in cold climates, such as in Saint-Genès-Champanelle and Velaine-en-Haye, climate change towards warmer temperatures could shift activity peaks towards early spring, and there would be no longer a winter pause; (2) in warm climates, such as in Carquefou and Gardouch, climate change towards even warmer and drier conditions could limit the distributions of suitable habitats that could support the physiological needs of *I. ricinus* ticks, i.e*.*, excessive thermal stress and difficulties in maintaining their water balance^7,8^.

Furthermore, we found that describing phenological patterns solely based on their shape, such as unimodal or bimodal, was insufficient. Rather, the timing of the phenological peak and the winter traits must be studied. To better understand the relationship between climatic gradient and phenological patterns, we recommend longitudinal studies conducted over large geographic areas to report the phenological pattern of each sampling location separately. Moreover, determining the age of nymphs through fat content analysis has been demonstrated to guide formulating hypotheses about the mechanisms for the population dynamics of each phenological pattern^17^.

As the computation for cumulated nymph counts using areas under the density curve^16^ requires strict, regular sampling intervals throughout the study period, we used annual maximum nymph counts as a proxy to explore inter-annual variations in questing nymph activity. Directional changes in the annual maximum nymph counts were observed only in Gardouch Outside but not Gardouch Inside, despite being located in the same area. We hypothesized that it could have occurred as a result of (1) a possible decrease in roe deer population outside of the experimental station, while the number of roe deer was always maintained at a higher inside the experimental station; or (2) the periods of heatwaves that impacted this area in summer 2019 and summer 2020 affected the tick survival, while the high host density in Gardouch Inside compensated for the tick mortality. Furthermore, a recent study suggested that the inter-annual fluctuation of nymph abundance could be explained by the level of tree seed production, and thus the abundance level of small mammal hosts, in previous years^59^. However, retrospective data on seed production were not available for all of our sampling sites during the study period, which precluded us from exploring this hypothesis in this study.

According to the regression analysis, the relationship between observed nymph counts (response variable) and interval-average meteorological variables was non-linear, indicating that the impacts on nymph counts were not the same at different levels of these meteorological variables. For example, when the 1-month average temperature ${T}_{M}^{0:1}$ was too high or too low, observed nymph counts decreased significantly, with a peak at an optimal value of ${T}_{M}^{0:1}$, which was dependent on the bioclimatic condition (Fig. 7A). Also, nymph counts were steadily low when the previous 3-to-6-month moving average temperature ${T}_{M}^{3:6}$ was greater than 15 °C, increased when ${T}_{M}^{3:6}$ was between 2 and 15 °C, and peaked at around 0–2 °C Fig. 7B). Finally, observed nymph counts dramatically increased when the 6-month moving average minimum relative humidity ${U}_{N}^{0:6}$ was greater than 90%, while nymph counts were relatively stable when ${U}_{N}^{0:6}$ was less than 90% (Fig. 7C).

As the number of nymphs collected in each sampling was a product of total population size of nymphs at the time of sampling (true abundance), the proportion of nymphs questing for a host, and the sampling rates, we hypothesized that the meteorological conditions influenced each of these unobserved parameters at different time lags. The population size is determined by the cumulative effects of past meteorological conditions, either directly (mortality rate or larva-to-nymph developmental rate) or indirectly (emigration/immigration from host movement). While the proportion of questing nymphs tended to respond to short-term fluctuation of the weather conditions^4^. A longitudinal observation on one site in Germany found similar effects of interval-average variables that explain the phenological pattern and inter-annual variation of *I. ricinus* activity^15^, which were current temperature ${T}_{M}^{0:0}$, previous 4-to-6-month average temperature ${T}_{M}^{4:6}$, and 1-month moving average relative humidity ${U}_{M}^{0:1}$. Furthermore, the best-fitted model also showed a significant interaction between the 1-month moving average temperature and a bioclimatic variable (average annual temperature ${BIO}_{Temp}$). This result indicated that although *I. ricinus* from colder climates start questing at lower temperature thresholds^57^, their activity arrives at its peak at higher temperatures (in summer; temperature ~ 14 to 16 °C). While tick activity in warmer climates peaks at lower temperatures (in spring; temperature ~ 11 to 14 °C). Presumably, the population size of questing nymphs in cold climates is unlikely to reach its peak during a colder spring due to developmental diapause (suppression of interstadial development of engorged ticks) or behavioural diapause (suppression of host-seeking behaviour activity of active ticks)^54,60^. In addition, photoperiod has also been proposed to regulate both behavioural and developmental diapause of ixodid ticks^54^; therefore, the exclusion of daytime duration from the best-fitted model, which was highly correlated with several meteorological variables, does not rule out the effects of photoperiod on tick activity dynamics.

The regression model also suggested that the baseline abundance level was associated with land cover characteristics. Sampling sites with a transition between moderately fragmented forest and non-forest areas, such as grassland or agricultural areas, support higher baseline nymph abundance (La Tour de Salvagny B, Gardouch Inside, Gardouch Outside, and Carquefou; ${p}_{Forest}$ ~ 17 to 52%). While landscapes with a high proportion of forest covers (${p}_{Forest}$ > 90%) tended to have intermediate (Velaine-en-Haye and Etiolles) or low (Les Bordes A to C, and Saint-Genès-Champanelle) baseline abundance level. These findings are consistent with previous studies^61–63^, indicating that forest fragmentation created transitional forest areas known as ecotones, which attract several mammal hosts^23^ and lead to a higher tick abundance. In addition, it has been suggested that forest fragmentation increases not only tick abundance but also the prevalence of pathogen-infected ticks^64^ and the risks of human-tick exposure^65^, which may lead to a higher incidence of tick-borne diseases. We also observed markedly low baseline *I. ricinus* abundance in the highly fragmented forest with a low proportion of forest-covering area (La Tour de Salvagny A; ${p}_{Forest}$ = 6.5%), possibly due to the lack of large foraging hosts in such a landscape^66^. Furthermore, we found a remarkably low baseline abundance in all three sampling sites in Les Bordes, which could not be fully explained biologically by our available environmental factors. This could be explained by the unsuitable conditions of Les Bordes forest, where superimposed layers of clay and sand lead to a temporary perched water table in winter, and the low soil water storage capacity leads to arid soil conditions in the upper parts of the soil profile during summer months with low rainfall. Other additional factors could also contribute to explain the low tick abundance at Les Bordes site such as host abundance, competition, or predation of a forest with such high biodiversity.

Desirable model diagnostic results and repeated resampling models using half of the original data produced similar estimate values over 500 iterations, indicating that all explanatory variables in the best-fitted model are robust. Model predictions also successfully predicted baseline abundance, phenological patterns, and inter-annual variations of *I. ricinus* nymph activity. However, the prediction from the best-fitted model still imperfectly captured the autumn peak in the phenological Pattern 2, where the sampling sites with this pattern had both fewer observations and lower abundance. This has brought us to the following inconclusive explanations: (1) the model failed to replicate the apparent autumn activity peak more clearly; (2) the apparent autumn peak was observed at random, and one additional nymph collected could generate a false signal when the sample size and baseline abundance were both small. As a result, it is envisioned that a more extended observation period and more sampling sites encompassing all phenological patterns should be implemented. Finally, although the regression analysis has been primarily used to find robust associations of environmental factors and help formulate ecological hypotheses on tick activity, it is considered a phenomenological model that does not fully explain the mechanisms of the observed phenomena^3^. In light of growing concerns about the impact of climate change on tick-borne diseases, particularly Lyme borreliosis, the mechanistic modelling framework for *I. ricinus* population dynamics is still required to investigate these ecological hypotheses.

## Conclusions

The study design in this study allowed us to longitudinally monitor the questing *I. ricinus* nymph activity, which is comparable across multiple tick observatories. Two distinct phenological patterns were identified in colder climates (a unimodal pattern with a summer peak and complete winter pause) and warmer climates (a unimodal pattern with an spring peak without winter pause). While a longer observation period is still required to confirm the presence of a spring–autumn bimodal phenological pattern in intermediate climates. Furthermore, the regression analysis revealed that phenological patterns and inter-annual variations in questing activity were associated with meteorological variables at different lag times. Our results also supported the notion that moderate forest fragmentation promotes higher nymph abundance. Our findings can guide formulating ecological hypotheses about the effects of climate change on the distribution, abundance, and phenology of this tick species, which can then be assessed using a mechanistic population dynamics model. Finally, the phenological patterns of *I. ricinus* activity predicted by our model could potentially be employed to develop tick-borne disease prevention tools, such as developing a seasonally dynamic human-tick exposure risk map, which could help to reduce Lyme borreliosis cases in France.

## Supplementary Information

Supplementary Information.

## Data Availability

The datasets generated during and/or analysed during the current study are available in the “TEMPO—Réseau National d'Observatoires de la Phénologie” repository, 10.15454/ZSYGUM.
