# Supplementary material for: Meteorological and climatic variables predict the phenology of Ixodes ricinus nymph activity in France, accounting for habitat heterogeneity
Source: Sci Rep. 2022 May 12;12:7833. doi: 10.1038/s41598-022-11479-z (PMC9098447; doi:10.1038/s41598-022-11479-z)
Supplement: Supplementary file 1 — Supplementary Information. [file 41598_2022_11479_MOESM1_ESM.docx]

# Supporting Information

# Meteorological and climatic variables predict the phenology of *Ixodes ricinus* nymph activity in France, accounting for habitat heterogeneity

Phrutsamon Wongnak, Séverine Bord, Maude Jacquot, Albert Agoulon, Frédéric Beugnet, Laure Bournez, Nicolas Cèbe, Adélie Chevalier, Jean-François Cosson, Naïma Dambrine, Thierry Hoch, Frédéric Huard, Nathalie Korboulewsky, Isabelle Lebert, Aurélien Madouasse, Anders Mårell, Sara Moutailler, Olivier Plantard, Thomas Pollet, Valérie Poux, Magalie René-Martellet, Muriel Vayssier-Taussat, Hélène Verheyden, Gwenaël Vourc’h, Karine Chalvet-Monfray


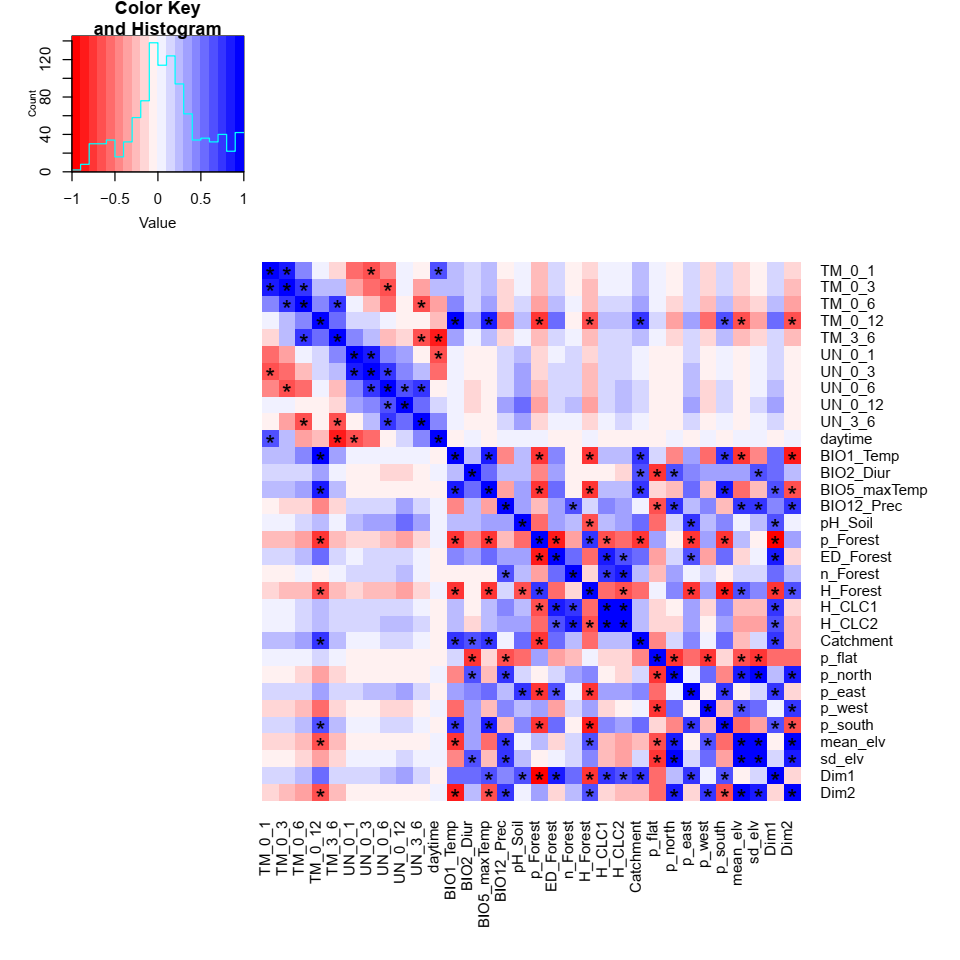


**Figure S1.** Pairwise correlation among environmental variables. Colours represent the value of Pearson’s correlation coefficient $r$, ranging from -1 (red) to 1 (blue) with asterisks indicating $\left| r \right|$ ≥ 0.6.


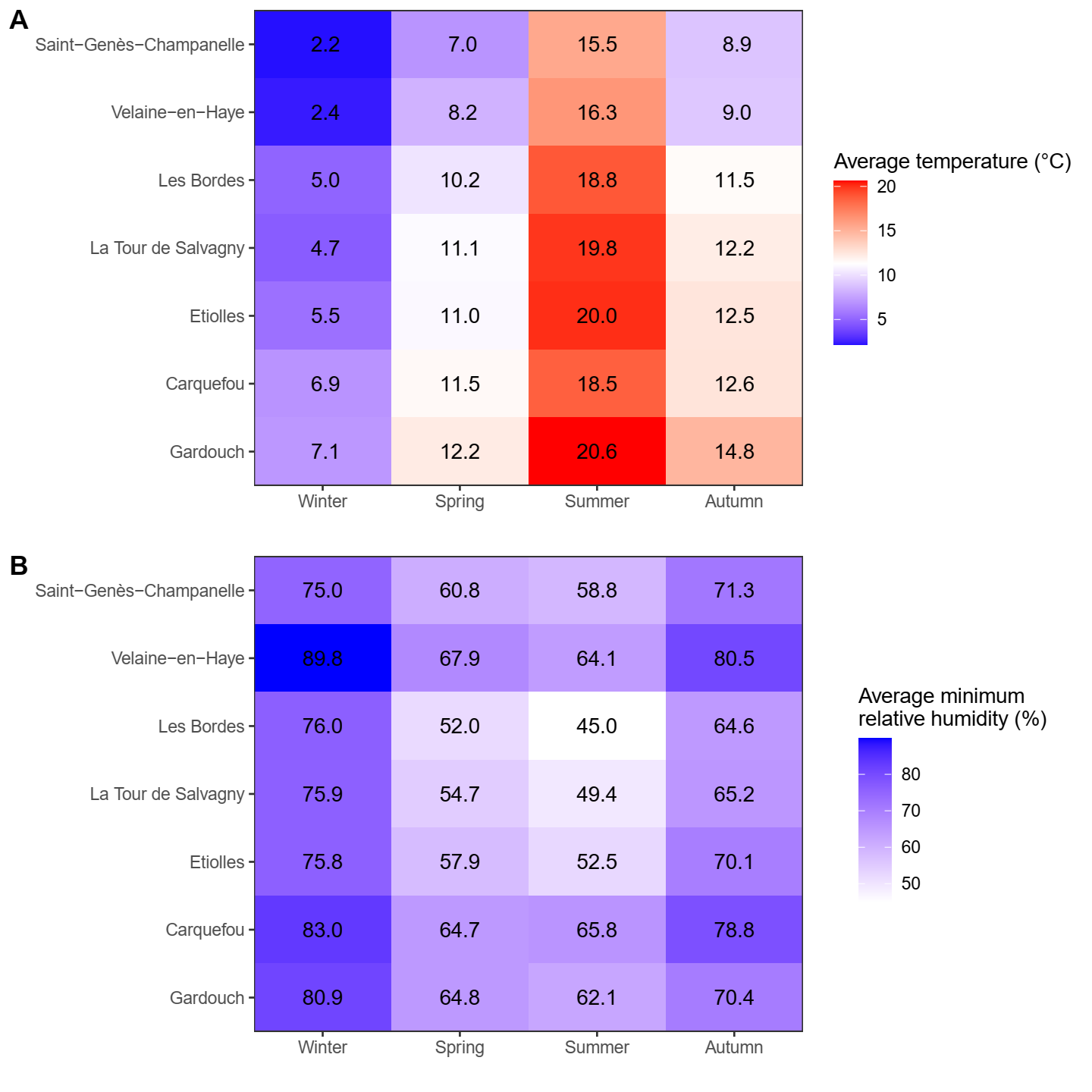


**Figure S2.** Seasonal average meteorological conditions of 7 tick observatories: A) Seasonal average temperature (°C); B) Seasonal average minimum relative humidity (%). Seasons were defined following meteorological seasons of the northern hemisphere: Winter, 1^st^ December to 28^th^ February; Spring, 1^st^ March to 31^st^ May; Summer, 1^st^ June to 31^st^ August; Autumn, 1^st^ September to 30^th^ November. Sites were arranged by the annual temperature ${BIO1}_{Temp}$, from Gardouch (high annual temperature) to Saint-Genès-Champanelle (low annual temperature).


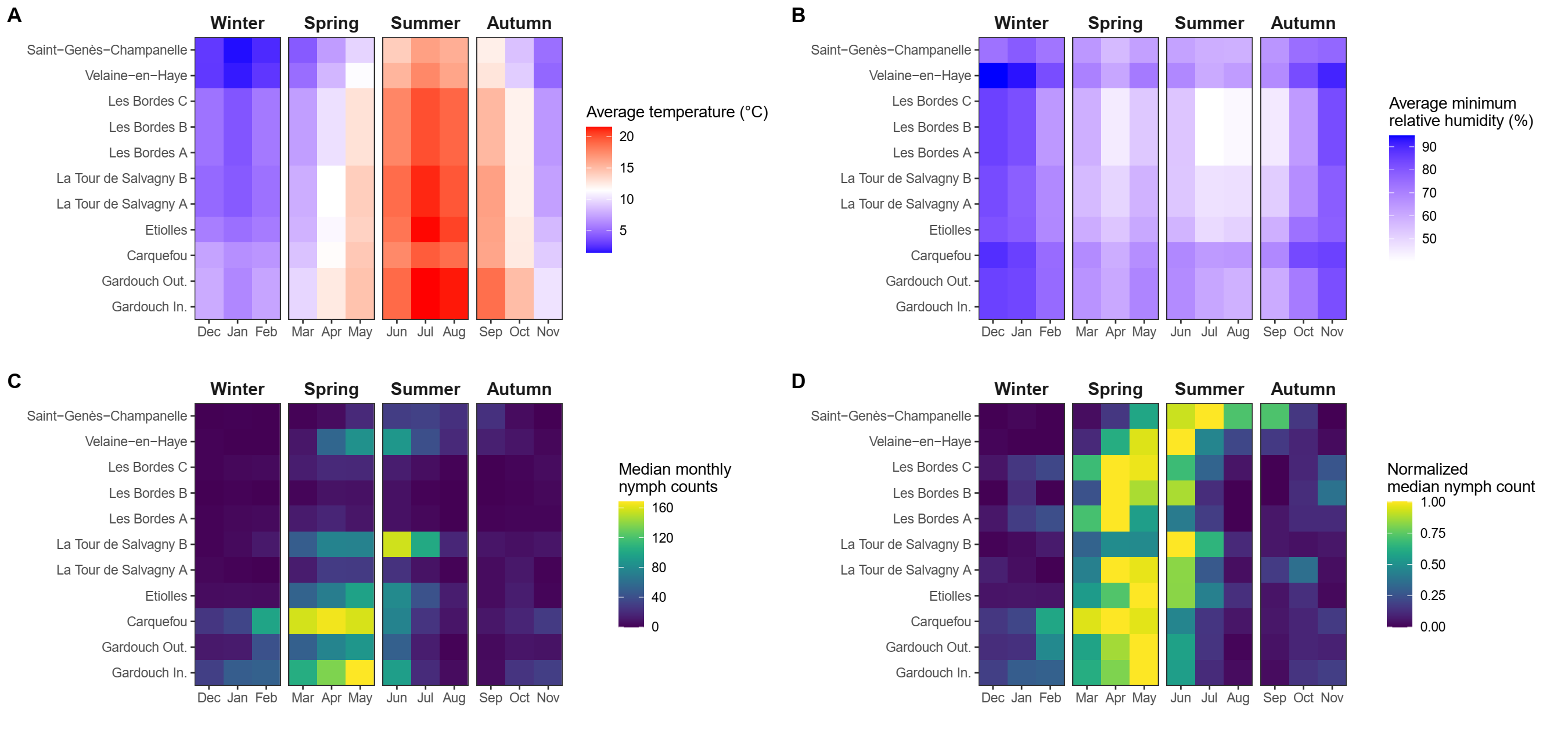


**Figure S3.** Monthly summary of nymph counts per 100 m^2^ and meteorological conditions of 11 sampling sites: A) Monthly average temperature (°C); B) Monthly average minimum relative humidity (%); C) Monthly median nymph counts per 100 m^2^; D) Normalized monthly median nymph counts per 100 m^2^. The value of 1 indicates the maximum nymph activity (peak), while the value of 0 indicates the absence of nymph activity. Facets were defined following meteorological seasons of the northern hemisphere: Winter, 1^st^ December to 28^th^ February; Spring, 1^st^ March to 31^st^ May; Summer, 1^st^ June to 31^st^ August; Autumn, 1^st^ September to 30^th^ November. Sites were arranged by the annual temperature ${BIO1}_{Temp}$, from Gardouch (high annual temperature) to Saint-Genès-Champanelle (low annual temperature).


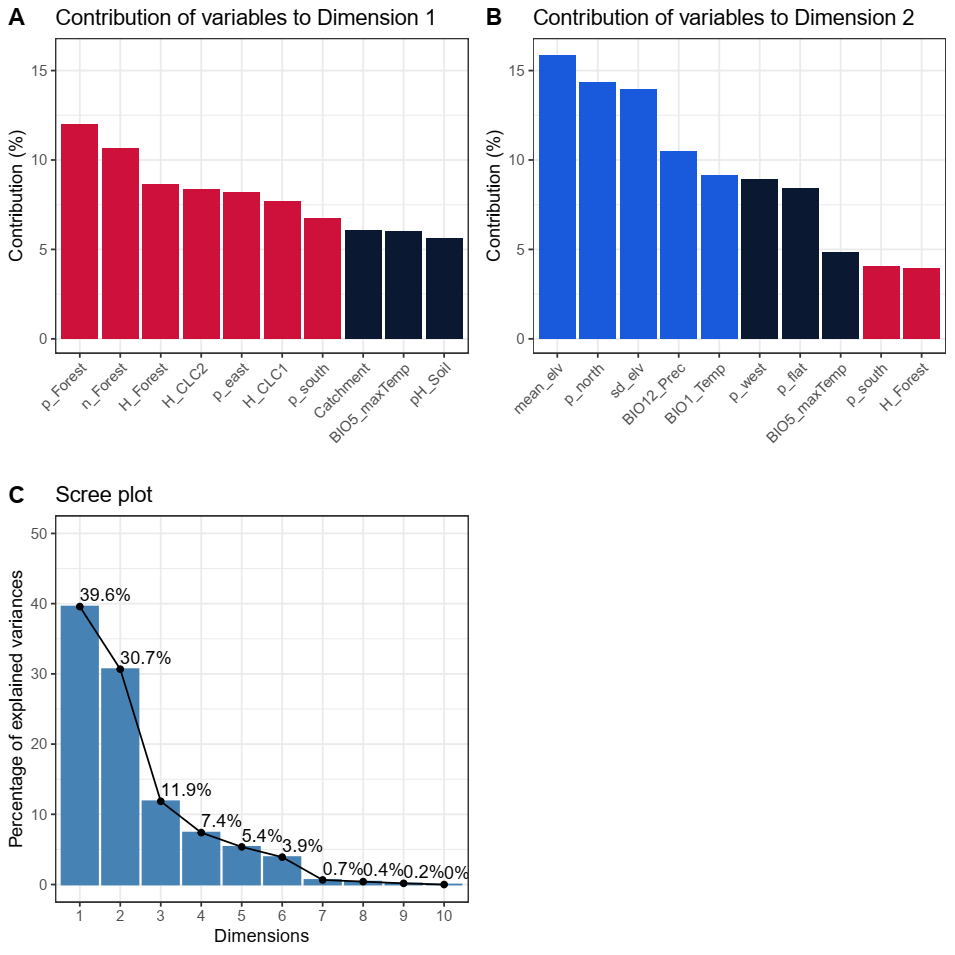


**Figure S4.** Details of the principal component analysis: Contributions of environmental variables on Dimension 1 (A), Dimension 2 (B), and the scree plot (C). Colours in panels A and B indicate whether the variable contributes more to Dimension 1 (red), Dimension 2 (blue), or other higher dimensions (black), using the cumulative contribution of ~60%.


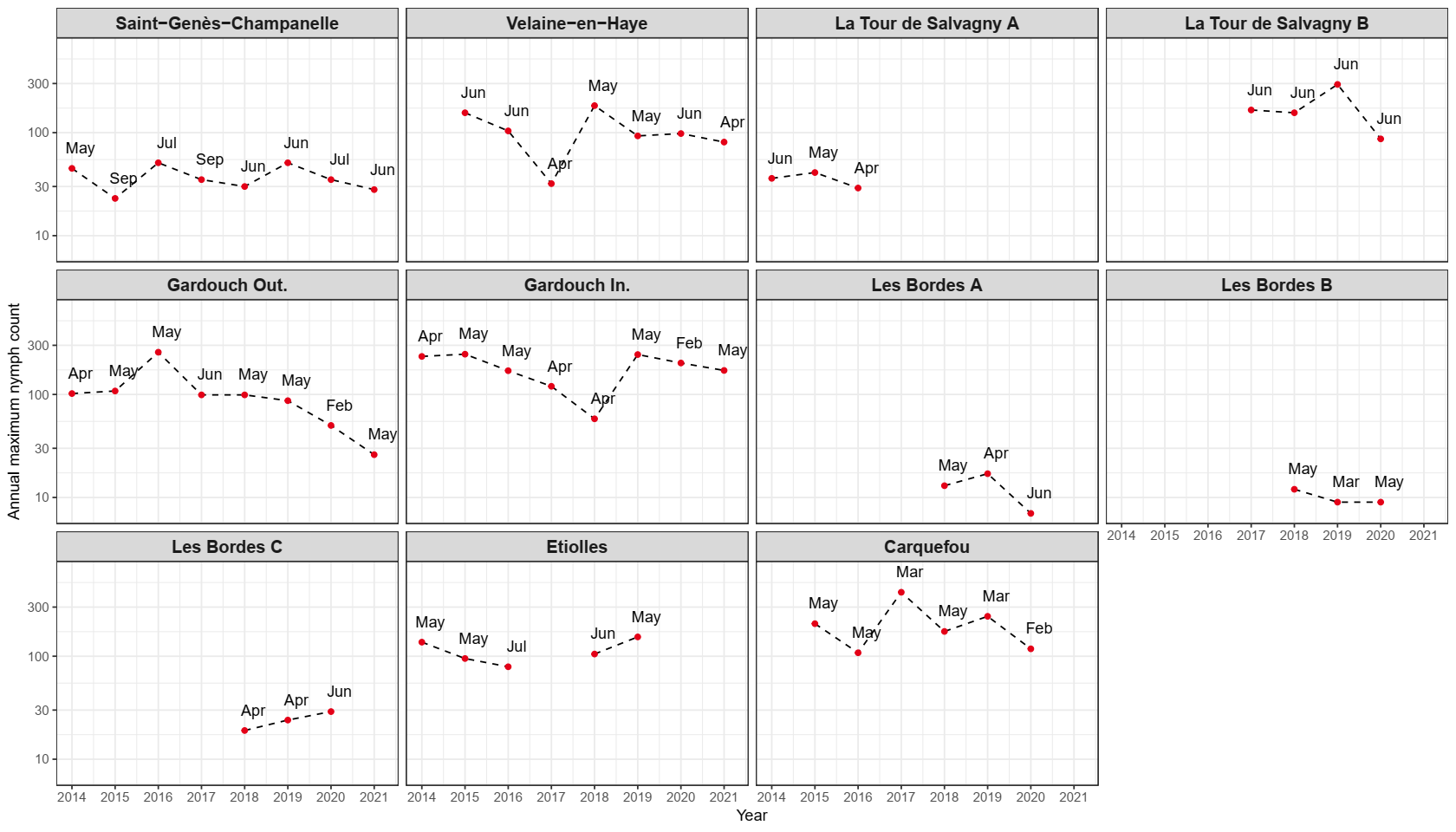


**Figure S5**.Trends of the annual maximum nymph counts per 100 m^2^ of 11 sampling sites. The maximum nymph counts of each site were display when the observation was carried out at least 1 campaign in spring (1^st^ March to 31^st^ May), and 1 campaign in summer (1^st^ June to 31^st^ August) of each year. Y-axes are represented on a logarithmic scale. Text annotations represent the month where the maximum nymph counts were observed.


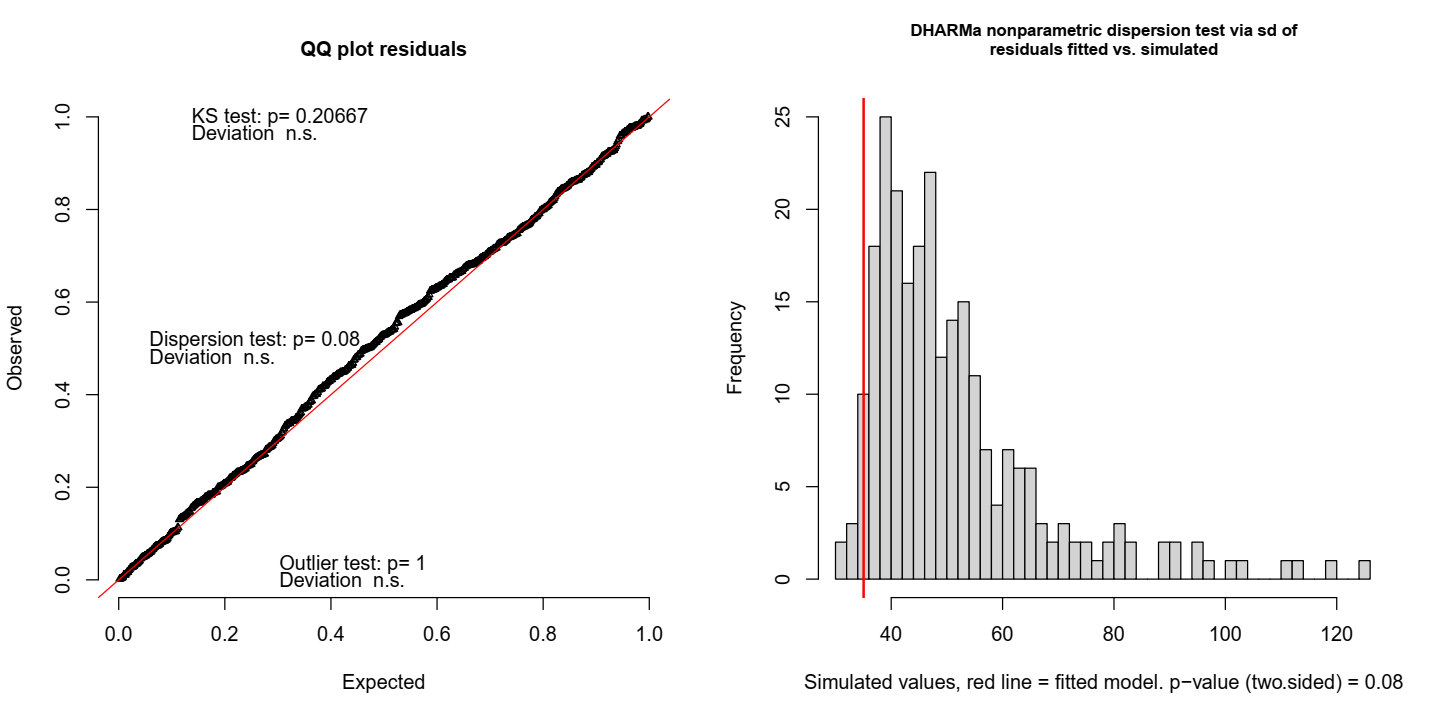


**Figure S6.** The model diagnostic for the best-fitted model. Left: Q-Q plot residuals with a Kolmogorov-Smirnov Goodness of Fit Test (K-S test), a dispersion test, and an outlier test. Right: a histogram of simulated dispersion test statistic values compared to the observed value from the fitted model (vertical red line).


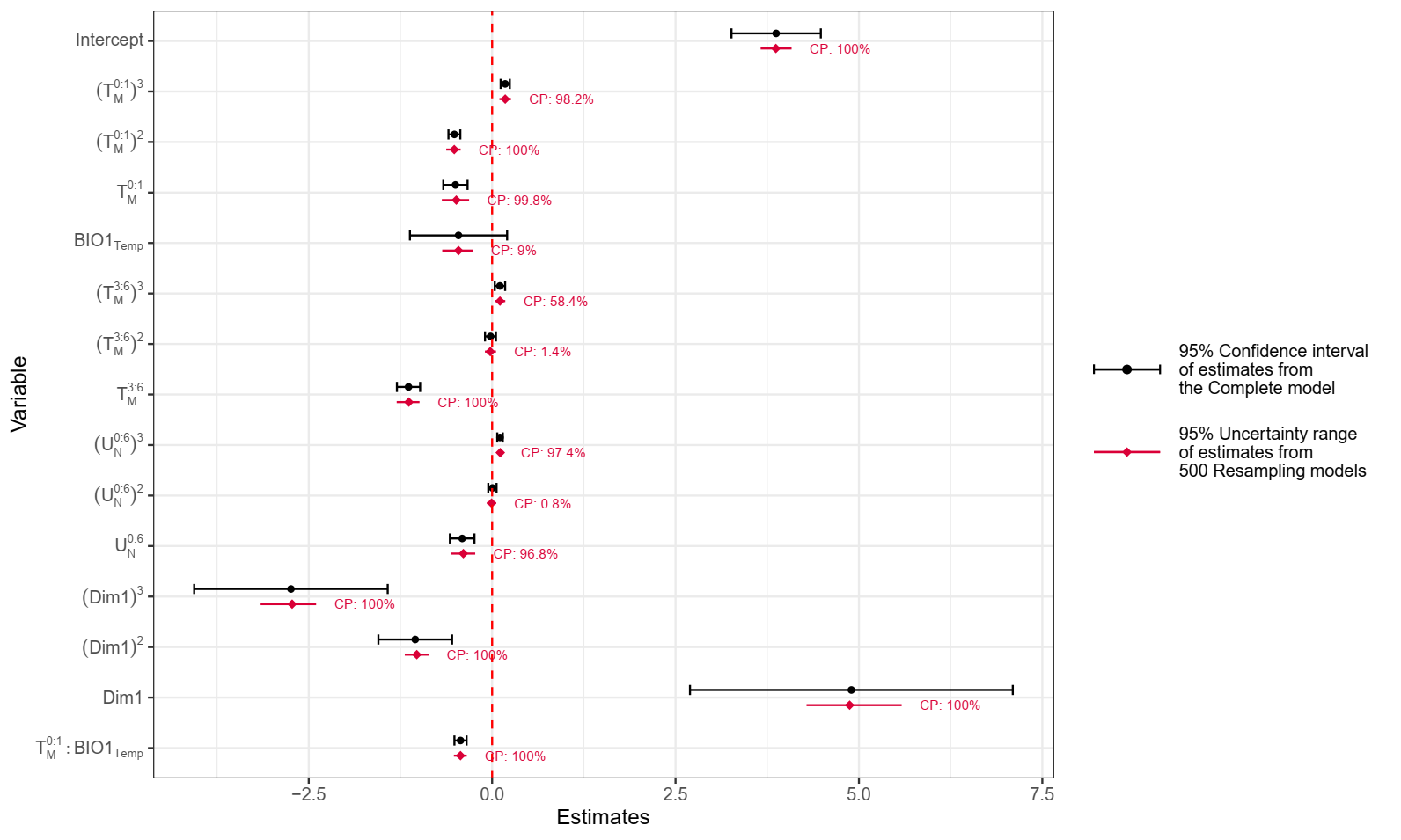


**Figure S7** Regression coefficients of environmental variables: One-month moving average temperature ($T_{M}^{0:1}$); Previous three-to-six-month moving average temperature ($T_{M}^{3:6}$); Annual temperature (${BIO1}_{Temp}$); Six-month moving average minimum relative humidity ($U_{N}^{0:6}$); Coordinates on Dimension 1 of the principal component analysis ($Dim1$); Interaction between $T_{M}^{0:1}$ and ${BIO1}_{Temp}$ ($T_{M}^{0:1}:{BIO1}_{Temp}$). The 95% confidence interval of estimates of the Complete model are represented in black, while the 95% uncertainty range of the estimates derived from 500 simulated resampling models are in red. The coverage probability (CP) shows the percentage of resampling models that produced estimates that were significantly different from zero ($p$ < 0.05). The dashed vertical line indicates the value zero.


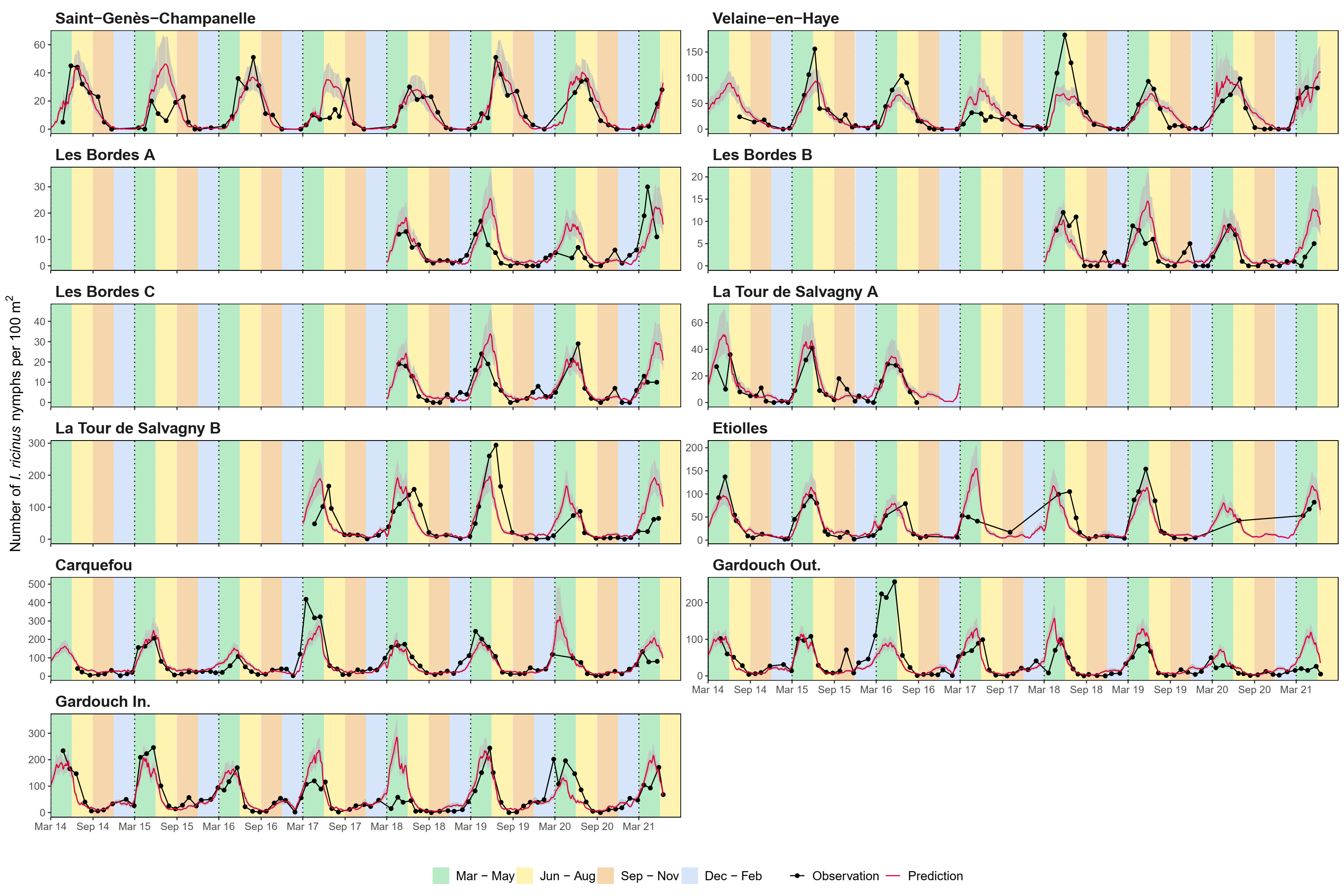


**Figure S8**. Comparison of *Ixodes ricinus* nymph counts per 100 m^2^ observed in the forests (black) and predicted from the best-fitted model (red). Gray shaded areas illustrate the 95% confidence interval of the prediction. Y-axes of different sampling sites were represented in different scales. Background colours indicate meteorological seasons of the temperate areas of northern hemisphere: Spring, 1^st^ March to 31^st^ May (green); Summer, 1^st^ June to 31^st^ August (yellow); Autumn, 1^st^ September to 30^th^ November (brown); Winter, 1^st^ December to 28^th^ or 29^th^ February (blue). Vertical dotted lines indicated 1-year interval.

**Table S1** Climate types, observation period, number of cloth-dragging campaigns, and nymph counts of 11 sampling sites.

| **Tick observatory** | **Climate type** | **Sampling site** | **Start** | **End** | **Campaigns** | **Nymphs** |
| --- | --- | --- | --- | --- | --- | --- |
| La Tour de Salvagny | Mixed | La Tour de Salvagny A | Apr 2014 | Sep 2016 | 29 | 343 |
|  |  | La Tour de Salvagny B | Apr 2017 | May 2021 | 43 | 2,443 |
| Saint-Genès-Champanelle | Mountain | Saint-Genès-Champanelle | Apr 2014 | Jun 2021 | 70 | 1,010 |
| Etiolles | Degraded oceanic | Etiolles | Apr 2014 | May 2021 | 52 | 2,090 |
| Carquefou | Oceanic | Carquefou | Jun 2014 | May 2021 | 82 | 5,454 |
| Gardouch | South-West Basin | Gardouch Inside | Apr 2014 | Jun 2021 | 86 | 5,452 |
|  |  | Gardouch Outside | Apr 2014 | Jun 2021 | 86 | 3,141 |
| Velaine-en-Haye | Semi-continental | Velaine-en-Haye | Apr 2014 | Jun 2021 | 72 | 2,391 |
| Les Bordes | Degraded-oceanic | Les Bordes A | Apr 2018 | May 2021 | 37 | 202 |
|  |  | Les Bordes B | Apr 2018 | May 2021 | 37 | 110 |
|  |  | Les Bordes C | Apr 2018 | May 2021 | 37 | 276 |

**Table S2** External meteorological data sources used as auxiliary variables in the imputation procedure.

| **Tick observatory** | **Source** | **Weather station** | **Postal code** | **Start** | **End** |
| --- | --- | --- | --- | --- | --- |
| La Tour de Salvagny | Météo-France | Brindas | 69028001 | Jan 2013 | Jun 2021 |
| Saint-Genès-Champanelle | INRAE | Saint-Genès-Champanelle | 63345002 | Jan 2013 | Jun 2021 |
| Etiolles | Météo-France | Orly | 91027002 | Jan 2013 | Jun 2021 |
| Carquefou | Météo-France | Nort-sur-Erdre | 44110002 | Jan 2013 | Jun 2021 |
| Gardouch | Météo-France | St. Felix Lauragais | 31478001 | Jan 2013 | Jun 2021 |
| Velaine-en-Haye | INRAE | Champenoux | 54113002 | Jan 2013 | Jun 2021 |
| Les Bordes | Météo-France | Amilly | 45004001 | Jan 2017 | Jun 2021 |

**Table S3** Land cover, forest characteristics, and soil pH of 11 sampling sites

| **Sampling site** | **Shannon’s index for CLC-1** | **Shannon’s index for CLC-2** | **Shannon’s index for forest** | **Forest-covering area (%)** | **Number of forest patches** | **Forest edge density (m/km^2^)** | **Soil pH** |
| --- | --- | --- | --- | --- | --- | --- | --- |
| La Tour de Salvagny A | 0.68 | 1.51 | 0.23 | 6.5 | 13 | 0.00281 | 5 |
| La Tour de Salvagny B | 1.00 | 1.53 | 0.89 | 52.1 | 9 | 0.00932 | 5 |
| Saint-Genès-Champanelle | 0.00 | 0.17 | 2.01 | 95.2 | 3 | 0.00408 | 5 |
| Etiolles | 0.00 | 0.00 | 1.05 | 99.6 | 1 | 0.00052 | 5 |
| Carquefou | 0.79 | 1.21 | 0.72 | 36.3 | 8 | 0.00626 | 5 |
| Gardouch Inside | 0.38 | 0.58 | 0.46 | 17.1 | 7 | 0.00320 | 6 |
| Gardouch Outside | 0.38 | 0.58 | 0.46 | 17.1 | 7 | 0.00320 | 6 |
| Velaine-en-Haye | 0.62 | 0.65 | 0.62 | 92.8 | 1 | 0.00399 | 6 |
| Les Bordes A | 0.10 | 0.10 | 1.48 | 98.7 | 1 | 0.00095 | 4 |
| Les Bordes B | 0.00 | 0.30 | 1.37 | 99.3 | 1 | 0.00062 | 4 |
| Les Bordes C | 0.00 | 0.00 | 1.22 | 99.3 | 1 | 0.00061 | 4 |

**Table S4** Topographic characteristics of 11 sampling sites

| **Sampling site** | **Catchment area** | **Mean elevation (m)** | **SD elevation (m)** | **Flat (%)** | **North (%)** | **East (%)** | **West (%)** | **South (%)** |
| --- | --- | --- | --- | --- | --- | --- | --- | --- |
| La Tour de Salvagny A | 7.58 | 316.9 | 12.4 | 11.8 | 17.1 | 32.1 | 18.9 | 20.1 |
| La Tour de Salvagny B | 8.67 | 292.4 | 11.5 | 11.6 | 22.5 | 21.0 | 30.9 | 14.1 |
| Saint-Genès-Champanelle | 7.49 | 989.9 | 54.7 | 7.1 | 32.0 | 21.8 | 30.9 | 8.2 |
| Etiolles | 7.51 | 89.1 | 1.9 | 37.0 | 11.9 | 20.9 | 18.8 | 11.4 |
| Carquefou | 7.90 | 8.8 | 3.1 | 33.0 | 15.2 | 22.5 | 15.6 | 13.7 |
| Gardouch Inside | 9.06 | 220.1 | 23.1 | 11.0 | 19.9 | 27.3 | 24.3 | 17.6 |
| Gardouch Outside | 9.06 | 220.1 | 23.1 | 11.0 | 19.9 | 27.3 | 24.3 | 17.6 |
| Velaine-en-Haye | 7.61 | 323.2 | 9.3 | 15.5 | 14.0 | 24.9 | 30.6 | 15.1 |
| Les Bordes A | 8.12 | 148.9 | 6.5 | 22.9 | 18.5 | 18.4 | 27.4 | 12.8 |
| Les Bordes B | 7.51 | 150.7 | 3.7 | 30.5 | 12.2 | 18.4 | 23.0 | 16.0 |
| Les Bordes C | 7.49 | 151.9 | 4.1 | 28.2 | 14.3 | 22.0 | 21.9 | 13.6 |

**Table S5** Bioclimatic characteristics of 11 sampling sites

| **Sampling site** | **Annual temperature (°C)** | **Diurnal range (°C)** | **Average temperature of the hottest month (°C)** | **Annual precipitation (mm)** |
| --- | --- | --- | --- | --- |
| La Tour de Salvagny A | 10.9 | 9.4 | 26.3 | 768 |
| La Tour de Salvagny B | 10.9 | 9.35 | 26.15 | 778 |
| Saint-Genès-Champanelle | 7.4 | 9.4 | 21 | 849 |
| Etiolles | 11.1 | 8.5 | 24.5 | 641 |
| Carquefou | 11.6 | 8.4 | 23.7 | 763.5 |
| Gardouch Inside | 12.75 | 9.65 | 26.9 | 740 |
| Gardouch Outside | 12.75 | 9.65 | 26.9 | 740 |
| Velaine-en-Haye | 9.2 | 8.5 | 23 | 767 |
| Les Bordes A | 10.9 | 9 | 24.55 | 668.5 |
| Les Bordes B | 10.9 | 9 | 24.575 | 665 |
| Les Bordes C | 10.9 | 9 | 24.575 | 665 |

**Table S6** Regression coefficients with the 95% confidence interval of the best-fitted model, and the coverage probability derived from 500 resampling models.

| **Variable** | **Estimate (95% confidence interval)** | **Coverage probability (%)** |
| --- | --- | --- |
| **Intercept** | 3.87 [3.26; 4.48] | 100.0 |
| **One-month moving average temperature:** |  |  |
| $(T_{M}^{0:1})^{3}$ | 0.18 [0.12; 0.24] | 98.2 |
| $(T_{M}^{0:1})^{2}$ | -0.51 [-0.60; -0.43] | 100.0 |
| $T_{M}^{0:1}$ | -0.50 [-0.67; -0.33] | 99.8 |
| **Previous three-to-six-month moving average temperature:** |  |  |
| $(T_{M}^{3:6})^{3}$ | 0.11 [0.04; 0.18] | 58.4 |
| $(T_{M}^{3:6})^{2}$ | -0.02 [-0.10; 0.05] | 1.4 |
| $T_{M}^{3:6}$ | -1.14 [-1.30; -0.98] | 100.0 |
| **Six-month moving average minimum relative humidity:** |  |  |
| $(U_{N}^{0:6})^{3}$ | 0.11 [0.07; 0.15] | 97.4 |
| $(U_{N}^{0:6})^{2}$ | 0 .00[-0.05; 0.06] | 0.8 |
| $U_{N}^{0:6}$ | -0.41 [-0.58; -0.24] | 96.8 |
| **Bioclimate:** |  |  |
| ${BIO1}_{Temp}$ | -0.46 [-1.12; 0.2] | 9.0 |
| **PCA Dimension 1:** |  |  |
| $(Dim1)^{3}$ | -2.74 [-4.06; -1.42] | 100.0 |
| $(Dim1)^{2}$ | -1.05 [-1.55; -0.55] | 100.0 |
| $Dim1$ | 4.90 [2.70; 7.10] | 100.0 |
| **Interaction of weather and climate:** |  |  |
| $T_{M}^{0:1}:{BIO1}_{Temp}$ | -0.43 [-0.51; -0.35] | 100.0 |
